# Subfamily logos: visualization of sequence deviations at alignment positions with high information content

**DOI:** 10.1186/1471-2105-7-313

**Published:** 2006-06-21

**Authors:** Eric Beitz

**Affiliations:** 1 Dept. of Pharmaceutical Chemistry, University of Tübingen, Morgenstelle 8, 72076 Tübingen, Germany

## Abstract

**Background:**

Recognition of relevant sequence deviations can be valuable for elucidating functional differences between protein subfamilies. Interesting residues at highly conserved positions can then be mutated and experimentally analyzed. However, identification of such sites is tedious because automated approaches are scarce.

**Results:**

Subfamily logos visualize subfamily-specific sequence deviations. The display is similar to classical sequence logos but extends into the negative range. Positive, upright characters correspond to residues which are characteristic for the subfamily, negative, upside-down characters to residues typical for the remaining sequences. The symbol height is adjusted to the information content of the alignment position. Residues which are conserved throughout do not appear.

**Conclusion:**

Subfamily logos provide an intuitive display of relevant sequence deviations. The method has proven to be valid using a set of 135 aligned aquaporin sequences in which established subfamily-specific positions were readily identified by the algorithm.

## Background

Most protein families can be divided into functionally distinct subfamilies. Such subfamilies exhibit characteristic properties which manifest for instance as binding specificity of regulatory proteins, substrate specificity of enzymes, and pore selectivity of channels and transporters. Functional differences are often linked to sequence characteristics in regions which are conserved throughout the protein superfamily. This is because conserved domains define the fold of the functional protein core or provide catalytic residues. Recognition of subfamily-specific deviations at such sites can be valuable for elucidating mechanistic principles of the protein family by site-directed mutagenesis and subsequent functional analysis of the mutants. An automated approach to identify relevant deviations should (i) provide the ability to take into account a large number of reference sequences, (ii) determine sequence conservation, i. e. positions of high information content, and (iii) visualize deviations, i.e. subfamily characteristics, relative to the information content in a graphical output which is easy to comprehend.

## Implementation

One sophisticated way of presenting sequence conservation is to display a sequence logo [6]. Here, the information content *I *(*P*_*i*_) of each alignment position *i *is defined inverse to the uncertainty *H*(*P*_*i*_) by the equation

I(Pi)=log⁡2|Σ|−H(Pi)=log⁡2|Σ|+∑j∈|Σ|Pij⋅log⁡2Pij
 MathType@MTEF@5@5@+=feaafiart1ev1aaatCvAUfKttLearuWrP9MDH5MBPbIqV92AaeXatLxBI9gBaebbnrfifHhDYfgasaacH8akY=wiFfYdH8Gipec8Eeeu0xXdbba9frFj0=OqFfea0dXdd9vqai=hGuQ8kuc9pgc9s8qqaq=dirpe0xb9q8qiLsFr0=vr0=vr0dc8meaabaqaciaacaGaaeqabaqabeGadaaakeaacqWGjbqscqGGOaakcqWGqbaudaWgaaWcbaGaemyAaKgabeaakiabcMcaPiabg2da9iGbcYgaSjabc+gaVjabcEgaNnaaBaaaleaacqaIYaGmaeqaaOGaeiiFaWNaeu4OdmLaeiiFaWNaeyOeI0IaemisaGKaeiikaGIaemiuaa1aaSbaaSqaaiabdMgaPbqabaGccqGGPaqkcqGH9aqpcyGGSbaBcqGGVbWBcqGGNbWzdaWgaaWcbaGaeGOmaidabeaakiabcYha8jabfo6atjabcYha8jabgUcaRmaaqafabaGaemiuaa1aaSbaaSqaaiabdMgaPjabdQgaQbqabaGccqGHflY1cyGGSbaBcqGGVbWBcqGGNbWzdaWgaaWcbaGaeGOmaidabeaakiabdcfaqnaaBaaaleaacqWGPbqAcqWGQbGAaeqaaaqaaiabdQgaQjabgIGiolabcYha8jabfo6atjabcYha8bqab0GaeyyeIuoaaaa@6858@

with |Σ| being the cardinality of the used alphabet, i.e. 4 for DNA and 20 for protein sequences, and *P*_*ij *_being the frequency of residue *j *at this position (variables according to [7]). Each position is displayed as a stack of residue symbols whose heights *l*_*ij *_represent their proportion of the information content:

*l*_*ij*_*= P*_*ij*_·*I*(*P*_*i*_)

Protein sequence logos are often adjusted to the background frequency of each amino acid in the alignment [7]. For simplicity, the variable name *I *(*P*_*i*_) will be used in the following for both, information content with or without frequency correction. Generally, both approaches are compatible with subfamily logos and have been implemented in the algorithm.

Contrary to a sequence logo that depicts sequence conservation, here, it is desired to display the relevance of deviations at conserved positions. The recently published pairwise HMM logo approach does align the sequence logos of two subfamilies [8]. This certainly facilitates the identification of relevant deviant positions, but one still has to inspect position by position and judge different symbol heights by eye. Subfamiliy logos provide a very intuitive display. They are derived by subtracting from the frequency *S*_*ij *_of a residue *j *within a pre-defined subset of sequences, i. e. a subfamily, the frequency *R*_*ij *_of this residue in the remaining set of sequences for each position *i*. The difference is then weighted by the overall information content *I*(*P*_*i*_) computed from all sequences and the residue is plotted with a symbol height of *s*_*ij*_:

*s*_*ij*_*= *(*S*_*ij*_*- R*_*ij*_)·fS˜R˜
 MathType@MTEF@5@5@+=feaafiart1ev1aaatCvAUfeBSjuyZL2yd9gzLbvyNv2Caerbhv2BYDwAHbqedmvETj2BSbqee0evGueE0jxyaibaiKI8=vI8tuQ8FMI8Gi=hEeeu0xXdbba9frFj0=OqFfea0dXdd9vqai=hGuQ8kuc9pgc9s8qqaq=dirpe0xb9q8qiLsFr0=vr0=vr0dc8meaabaqaciGacaGaaeqabaqadeqadaaakeaacaWGMbWaaSbaaSqaaiqadofagaacaiqadkfagaacaaqabaaaaa@3611@·*I*(*P*_*i*_)

The term (*S*_*ij*_*- R*_*ij*_) gives values from -1 to 1. Positive values correspond to residues which are characteristic for the subfamily (shown upright in the output), negative values to those that are typical for the remaining sequences (shown upside-down). Positions with an equal distribution of residue *j *result in a zero value.

The need for a correction factor fS˜R˜
 MathType@MTEF@5@5@+=feaafiart1ev1aaatCvAUfeBSjuyZL2yd9gzLbvyNv2Caerbhv2BYDwAHbqedmvETj2BSbqee0evGueE0jxyaibaiKI8=vI8tuQ8FMI8Gi=hEeeu0xXdbba9frFj0=OqFfea0dXdd9vqai=hGuQ8kuc9pgc9s8qqaq=dirpe0xb9q8qiLsFr0=vr0=vr0dc8meaabaqaciGacaGaaeqabaqadeqadaaakeaacaWGMbWaaSbaaSqaaiqadofagaacaiqadkfagaacaaqabaaaaa@3611@ is illustrated by the following example. Assume an alignment with an equal number of sequences in the subfamily and in the remaining set of sequences. Further, assume a position *i *within the alignment where all sequences in the subfamily carry amino acid *a *and all remaining sequences carry amino acid *b *with *a *≠ *b*. This situation can be considered as the best possible discrimination between the subfamily and the remaining set of sequences and results in the frequencies *P*_*ia *_= 0.5, *P*_*ib *_= 0.5 and all other *P*_*ij *_= 0. The overall information content at this position, thus, is *I*(*P*_*i*_) = Iog_2 _20 + 0.5 Iog_2 _0.5 + 0.5·Iog_2_·0.5 = Iog_2 _20 - 1, i. e. one bit less than the maximal information content. For either group of sequences, however, the information content should be maximal due to the frequencies *S*_*ia*_*= *1 (subfamily) and *R*_*ib *_= 1 (remaining sequences). The decrease in the apparent information content depends on the fraction of sequences in the subfamily (S˜
 MathType@MTEF@5@5@+=feaafiart1ev1aaatCvAUfKttLearuWrP9MDH5MBPbIqV92AaeXatLxBI9gBaebbnrfifHhDYfgasaacH8akY=wiFfYdH8Gipec8Eeeu0xXdbba9frFj0=OqFfea0dXdd9vqai=hGuQ8kuc9pgc9s8qqaq=dirpe0xb9q8qiLsFr0=vr0=vr0dc8meaabaqaciaacaGaaeqabaqabeGadaaakeaacuWGtbWugaacaaaa@2DEA@) and in the remaining set (R˜
 MathType@MTEF@5@5@+=feaafiart1ev1aaatCvAUfKttLearuWrP9MDH5MBPbIqV92AaeXatLxBI9gBaebbnrfifHhDYfgasaacH8akY=wiFfYdH8Gipec8Eeeu0xXdbba9frFj0=OqFfea0dXdd9vqai=hGuQ8kuc9pgc9s8qqaq=dirpe0xb9q8qiLsFr0=vr0=vr0dc8meaabaqaciaacaGaaeqabaqabeGadaaakeaacuWGsbGugaacaaaa@2DE8@). Hence, the factor fS˜R˜
 MathType@MTEF@5@5@+=feaafiart1ev1aaatCvAUfeBSjuyZL2yd9gzLbvyNv2Caerbhv2BYDwAHbqedmvETj2BSbqee0evGueE0jxyaibaiKI8=vI8tuQ8FMI8Gi=hEeeu0xXdbba9frFj0=OqFfea0dXdd9vqai=hGuQ8kuc9pgc9s8qqaq=dirpe0xb9q8qiLsFr0=vr0=vr0dc8meaabaqaciGacaGaaeqabaqadeqadaaakeaacaWGMbWaaSbaaSqaaiqadofagaacaiqadkfagaacaaqabaaaaa@3611@ was introduced, which follows the form shown in the example above and corrects for the described error:

fS˜R˜=log⁡2|Σ|log⁡2|Σ|+S˜⋅log⁡2S˜+R˜⋅log⁡2R˜
 MathType@MTEF@5@5@+=feaafiart1ev1aaatCvAUfKttLearuWrP9MDH5MBPbIqV92AaeXatLxBI9gBaebbnrfifHhDYfgasaacH8akY=wiFfYdH8Gipec8Eeeu0xXdbba9frFj0=OqFfea0dXdd9vqai=hGuQ8kuc9pgc9s8qqaq=dirpe0xb9q8qiLsFr0=vr0=vr0dc8meaabaqaciaacaGaaeqabaqabeGadaaakeaacqWGMbGzdaWgaaWcbaGafm4uamLbaGaacuWGsbGugaacaaqabaGccqGH9aqpdaWcaaqaaiGbcYgaSjabc+gaVjabcEgaNnaaBaaaleaacqaIYaGmaeqaaOGaeiiFaWNaeu4OdmLaeiiFaWhabaGagiiBaWMaei4Ba8Maei4zaC2aaSbaaSqaaiabikdaYaqabaGccqGG8baFcqqHJoWucqGG8baFcqGHRaWkcuWGtbWugaacaiabgwSixlGbcYgaSjabc+gaVjabcEgaNnaaBaaaleaacqaIYaGmaeqaaOGafm4uamLbaGaacqGHRaWkcuWGsbGugaacaiabgwSixlGbcYgaSjabc+gaVjabcEgaNnaaBaaaleaacqaIYaGmaeqaaOGafmOuaiLbaGaaaaaaaa@5B33@

## Results and discussion

**Figure 1:**
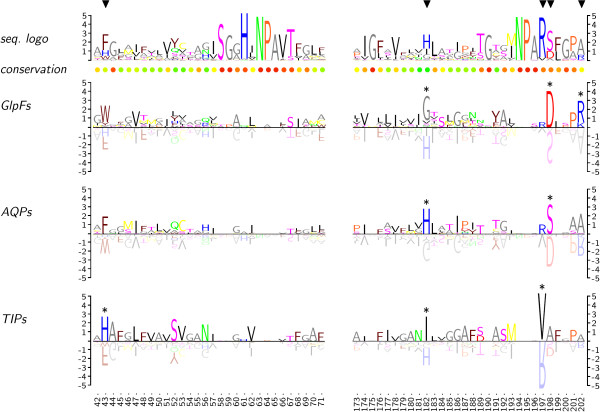
**Subfamily logos in comparison to classical sequence logos**. Sections of three aquaporin subfamilies are shown, i.e. water/glycerol channels (GlpFs), water-specific channels (AQPs), and tonoplast intrinsic proteins (TIPs). Subfamily-specific residues are displayed upright, residues that are typical for the remaining sequences as tinted upside-down characters. The unit of the ordinates is in bits. Triangles mark known positions of relevant subfamily-specific deviations. Asterisks were computed by the subfamily logo algorithm to label subfamily-specifc residues.

Fig. 1 displays two sections of a protein alignment (pos. 42–71 and 173–202) which consists of a total of 135 aquaporin sequences. Two functionally distinct subfamilies are represented by 32 aquaglyceroporins (GlpFs; permeability for water and glycerol), and 103 water-specific aquaporins (AQPs). From the latter, another water-specific subfamily consisting of 11 plant tonoplast intrinsic proteins (TIPs) can be separated.

The frequency-corrected sequence logo on the top highlights conserved positions around the two canonical Asn-Pro-Ala (NPA) motifs. The scale of the ordinate is in bits. Sequence conservation is further indicated by a color scale below the logo based on a structural matrix integrated into TEXshade. The triangles mark positions where the GlpF, AQP, and TIP subfamilies deviate as shown before in various publications [2,4,5]. These positions are directly connected to function because they contribute to the layout of the selective pore constriction.

Three frequency-corrected subfamily logos are shown below. Readability is greatly improved when upside-down symbols are tinted by 50%. This gives the impression of a reflective surface with a focus on the positive, subfamily-relevant residue symbols. The output is intuitive and basically self-explaining. Positions which are conserved throughout do not appear in the subfamily logos, see for instance the NPA motifs at positions 63–65 and 194–196. However, sequence deviations become visible dependent on the information content, e.g. Val_197 _vs. Arg in the TIP subfamily, or Asp_198 _and Ser_198_, respectively, in the GlpF or AQP subfamilies. Deviations are less pronounced at positions with a higher number of possible residues due to the lower information content. Nevertheless, subfamily characteristics are still visible if relevant, e.g. at positions 43, 182, and 202. The algorithm further accepts a threshold bit-value above which a deviant residue is additionally highlighted by a symbol (asterisks in Fig. 1). Empirically, this value is set to *log*_2_5 (2.322 bit) for proteins, which corresponds to the presence of one particular residue in 25% of all sequences or 50% of the subfamily, and *log*_2_2 (1 bit) for DNA sequences. The threshold value can be manually adjusted to match the alignment situation in question. It may also be used in the future to indicate statistical evaluations of the residue distribution. Inherently, best results are obtained when only two subfamilies are compared.

**Figure 2:**
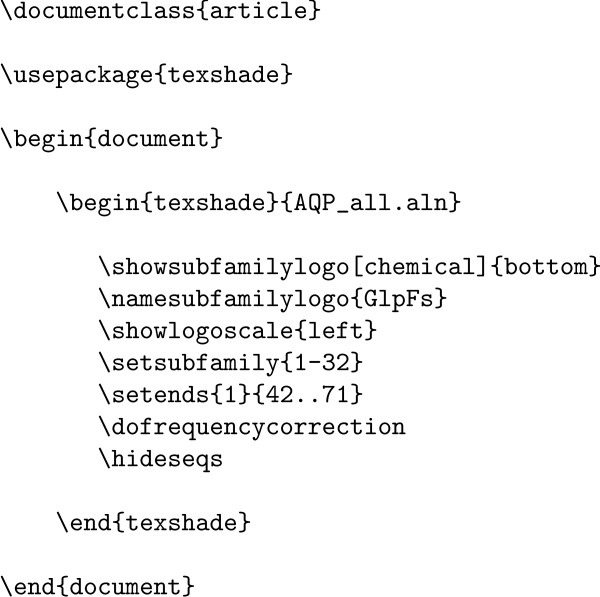
**Example input for subfamily logo generation**. Shown is the code needed to calculate and display positions 42–71 of the subfamily logo for the GlpF aquaporin subfamily displayed in Fig. 1. The input file AQP_all.aln contains a multiple sequence alignment of 135 aquaporin protein sequence.

Currently, subfamily logos are implemented in TEXshade [see additional files 1 and 2], i.e. a LATEX macro package for setting and shading multiple sequence alignments [1]. Some sample code is displayed in Fig. 2 depicting that a small number of commands leads to satisfying output. TEXshade provides numerous additional commands for individual adjustments of the output and comprehensive labeling. However, implementation of a subfamily logo extension into software that provides a graphical user interface and TEXshade output, such as STRAP [3] or the San Diego Supercomputer Center Biology WorkBench , is strongly encouraged. Further, integration of the subfamily logo algorithm into local or web-based sequence logo plotting tools should be straight forward.

## Conclusion

Subfamily logos are an extension to the classical application of sequence logos. They provide a novel tool to intuitively visualize subfamily sequence characteristics. The validity of the method was confirmed by analysis of 135 aligned aquaporin sequences and correct identification of subfamily-specific sequence deviations. Their relationship to sequence logos makes it easy to integrate them into existing logo software.

## Availability and requirements

Project name: TEXshade

Project home page:  or any CTAN site

Operating system(s): Platform independent

Programming language: LATEX

Other requirements: LATEX2ε

License: GNU GPL

Any restrictions to use by non-academics: none

## Authors' contributions

EB designed, implemented, and tested the algorithm and prepared the manuscript.

## Supplementary Material

Additional file 1 TEXshade source code. This is the TEXshade macro package containing the sequence logo algorithm and documentation as a LATEX docstrip archive Click here for file

Additional file 2 LATEX instruction file. This file contains the LATEX instructions for unpacking the TEXshade macros Click here for file
